# Exploring adherence to antihypertensive medication in Black African and Black Caribbean residents in South East London: a qualitative study

**DOI:** 10.3399/BJGPO.2024.0127

**Published:** 2025-12-19

**Authors:** Nupur Yogarajah, Kathryn Griffiths, Kate Bramham, Amy Baraniak

**Affiliations:** 1 Clinical lead for population health and inequalities (Greenwich), General Practitioner, NHS South East London integrated care board, University of Derby, Derby, United Kingdom; 2 Renal research associate & Health inequalities fellow in Population health, King’s College London, Lewisham and Greenwich population health and care team (Lewisham and Greenwich NHS Trust), London, United Kingdom; 3 Reader of Nephrology & Maternal Medicine and Honorary Consultant Nephrologist, King’s College London, King’s College Hospital, London, United Kingdom; 4 Health Psychologist, Subject Lead Psychology, University of Derby, Derby, United Kingdom

**Keywords:** hypertension, inequalities, ethnic groups, ethnicity

## Abstract

**Background:**

Hypertension disproportionately affects individuals identifying as Black African, Black Caribbean, and Black other with higher rates of uncontrolled hypertension and related organ damage including stroke and kidney disease. Improved understanding of ethnic and cultural views about hypertension is needed to support medication adherence.

**Aim:**

To explore the adherence barriers and facilitators to taking antihypertensive medication in people of Black African or Black Caribbean heritage.

**Design & setting:**

Qualitative study with an electronic survey followed by semi-structured interviews in South East London.

**Method:**

11 participants were recruited with the support of public health and a community interest company; nine who completed an online survey and six who completed online interviews, regarding their experiences and thoughts around medical management of hypertension. Data were analysed using thematic analysis and then mapped to Capacity, Opportunity, and Motivation Behaviour (COM-B) model components.

**Results:**

Substantial barriers exist to the adherence to antihypertensive medication for Black African and Black Caribbean patients in South East London owing to mistrust on both system and interpersonal levels. People felt uninvolved in treatment decisions and that there was a lack of discussion about non-medical management. Adherence was facilitated by an understanding of the consequences of not taking antihypertensive medication, although this was also associated with fear and mistrust.

**Conclusion:**

These barriers add new findings to existing studies on hypertension management and are congruent with current literature describing mistrust stemming from longstanding racial discrimination. Mapping to a COM-B model allows clinicians, and wider systems, to translate these findings into opportunities for interventions. Recommendations include patient-centred consultations to improve health literacy and shared decision making, trust-based engagement with communities and cultural awareness training.

## How this fits in

This study explores the relationship between ethnic identity and medical adherence in the context of hypertension in a diverse urban setting in South East London. It goes beyond current existing literature by mapping findings to a behavioural model (Capacity, Opportunity, and Motivation Behaviour; COM-B), which allows possible solutions and tools for clinicians to take on in their day-to-day practice as well as for health systems as a whole to consider, such as cultural humility training and adopting shared decision making, in the primary care setting. The emergence of trust-based barriers identified in this study are indicative of deep-rooted influencers on aspects of health behaviours critical to tackling broad inequalities, as described by others, implying the need for all healthcare professionals to be acknowledging, cognisant, and understanding of these wider historical issues, which could be playing a role in multiple aspects of health care. These issues need to be urgently addressed to improve health equity in hypertension management and beyond.

## Introduction

There is an urgent need to improve hypertension care from case finding to clinical management; a study in 2019 reviewed more than 320 000 primary care records of multi-ethnic adults aged 18–80 years and found approximately half of those expected to have hypertension were diagnosed, and of these, only half had adequate blood pressure control.^1^ In Lambeth, South East London, a review of 320 094 patient records showed that Black ethnicity was a predictor of uncontrolled hypertension compared with the White British population, the adjusted odds ratio (aOR) for the Black African population was 1.39 (95% confidence interval [CI] = 1.25 to 1.53) and for the Black Caribbean was 1.31 (95% CI = 1.19 to 1.45). This is supported by a study looking at hypertension in participating adults aged 40–69 years through the UK Biobank, which described reduced odds of having controlled hypertension for those with recorded Black ethnicity (OR 0.73, 95% CI = 0.65 to 0.82 compared with White) and this was in a population with 92.9% White ethnicity.^2^

It is essential to view these poorer health outcomes within the broader social determinants of health. The *Build Back Fairer: COVID-19 Marmot Review* ^2^ brought to the fore ethnic inequalities in raised mortality risk with COVID-19 infection in Black, Asian, and minority ethnic groups compared with White individuals in England, owing to cumulative risk collection of living in more deprived areas, having occupations with greater levels of proximity to other people, and more long-term conditions (LTCs).

Estimates suggest that up to half the medications prescribed for LTCs, such as hypertension, are not taken as advised,^3^ and in 2010 the gross annual cost to the NHS of primary and community care medicines wastage was approximately £300 million, accounting for £1 in every £25 spent in this area.^4^ A systematic review of qualitative research on lay perspectives of hypertension and medication adherence focusing on 53 studies, including all ethnic groups across 16 countries (including the UK), identified patients understanding of the causes and effects of their hypertension was critical in explaining non-adherence to medication, with common themes of believing hypertension caused symptoms such as headaches and dizziness and therefore absence of these symptoms or triggers precluded need to take antihypertensive drugs.^5^ This contrasts with medical and scientific communities describing hypertension as a predominantly asymptomatic condition^6^ requiring long-term daily medication.^3^ These paradoxical patient and medical explanations of hypertension accentuate the importance of conducting qualitative studies to understand patient perspectives of raised blood pressure and medications used to treat the condition, to enable appropriate intervention design to improve adherence and subsequently lower the risk of severe complications.

The Capacity, Opportunity, and Motivation Behaviour model (COM-B)^7^ underpins the behaviour change wheel (BCW) framework that can guide evidence-based intervention design.^8^ By understanding the three components of a behaviour (capability, opportunity, and motivation) it can be seen why a specific behaviour, in this case non-adherence to antihypertensive medication, occurs and thus generate targeted interventions that may alter this behaviour.

In Greenwich, South East London, 21% of residents identified as Black, Black British, Black Welsh, Caribbean or African in the 2021 census,^9^ making it a relevant setting to investigate barriers and facilitators to blood pressure management for Black patients; the participation of a local public health and community interest company (CIC), Mabadiliko, facilitated a connection with residents to invite them for interview.

Our study set out to explore adherence to hypertension medication in Black African and Black Caribbean residents in an urban area with significant deprivation, health inequalities and a rising challenge for medical teams to meet the needs of their population.^3^ The analysis and mapping to COM-B behavioural model set out to propose pragmatic actions for GPs and primary care as a whole to adopt.

## Method

Adults aged ≥18 years, residing in South East London, identifying as Black African, Black Caribbean, or Black Other, who had been advised they have high blood pressure and had been offered or prescribed medication for hypertension by a UK-based healthcare professional (HCP), were eligible for inclusion. This study was initially designed as an online survey to improve accessibility but there was a relatively poor response. A decision was made to purposivefully sample and this was enabled through recruitment via Public Health in Royal Borough of Greenwich (PHRBG), and Mabadiliko, a CIC; both organisations are based in South East London and hold distribution networks to the participants of interest. PHRBG circulated the advert via its community champions and neighbourhood coordinators' WhatsApp groups, and Mabadiliko used email, WhatsApp groups, and a direct approach to individuals across its network.

The interviews were conducted by the first author (NY), who is a GP in South East London, as part of an MSc in behavioural science. Thematic analysis was conducted from an essentialist approach to comprehend participant beliefs and experiences at face value and processed in a structured format following defined analytic phases as detailed below.^10^

Data were analysed by NY who is also clinical lead for quality improvement in South East London and a second-generation South-Asian immigrant. Input regarding methodology, reviewing themes, and report writing was provided by the co-authors with relevant academic and/or clinical expertise (University of Derby, King’s College London). Data saturation was considered reached when no new information was being generated that contributed to coding or theme development or ‘informational redundancy' as opposed to theoretical saturation.^11^

### Collecting data

Participants were sent an online survey, which contained all materials including study information, consent, questions set to support data collection, and debrief at the end. Initial questions focused on collection of demographic details including ethnicity, age, and sex, and ensuring inclusion criteria were met. Open-ended questions followed to broadly explore barriers and facilitators based on the constructs of COM-B ( Table 1).^7^ Following the debriefing, a final question appeared establishing willingness to be interviewed on the same topic. If the participant indicated positively, this led to another page to securely provide their contact email and/or phone number and they were advised contact would be made within 6 weeks. If they indicated negatively, they were thanked, and the survey terminated.

**Table 1.** Sample questions

Topic	Sample questions and prompts
1. Experience of having hypertension	Could you tell me a little bit about your experiences of having high blood pressure please?
	When were you diagnosed?
	How did you react to your diagnosis?
2. Views on blood pressure medication	What are your views about blood pressure medication?
	Were blood pressure medications prescribed at the point of diagnosis?
	How did you feel about the prescribed medications?
	How was the experience of taking medication?
3. Managing blood pressure	Do you use other strategies to control your blood pressure or the effects of your blood pressure medication?
	Please describe any factors that help you take your medication
	Please describe any challenges that make taking your medication difficult
	Please describe anything else you feel is important that we haven’t covered today

The interview schedule followed the same format as the survey and were semi-structured in nature; sample questions are outlined in table 1. Interviews were conducted via Microsoft Teams or phone, were recorded and transcribed verbatim, and stored securely under a unique code generated by the participant.

### Generating initial codes and searching for themes

All codes were generated manually without use of software, through in-depth analysis of the verbatim transcriptions. Codes were created from information in the data relevant to the research question and once all data were coded, broader themes emerged, which encapsulated several codes within them.

### Reviewing themes

Last author (AB) who is a health psychologist and senior lecturer reviewed the themes.

### Defining and naming themes

Following this inductive stage, the themes were then deductively mapped onto the COM-B framework to delineate their nature into capability, opportunity, or motivational antecedents of behaviour and hence barriers or facilitators to the desired behaviour. Once themes were generated, they were analysed deductively to understand which constructs of the COM-B model they were aligned to.

### Report writing

Synthesising the report and consideration of findings within current literature, as well as how it may lead to changes in clinical practice and health system response, were done collectively by all authors with KG and KB having considerable experience in culturally tailored programmes of care for hypertension and kidney disease.

## Results

Of 29 returned surveys, 20 were excluded owing to not meeting inclusion criteria (*n* = 6) or incomplete response (*n* = 14), leaving nine usable surveys. Survey responders ranged in age from 48–78 years, with an average age of 59 years. Seven survey responders consented to be contacted for interview, of which two did not respond and one withdrew, leaving four who were interviewed. An additional two interviewees were recruited opportunistically through Mabadiliko and hence did not complete the survey beforehand.

The six interviewees ranged in age from 53–65 years, with an average age of 59 years. Interviews ranged in duration from 18–51 minutes, with an average length of 36 minutes. Demographic characteristics of all participants are summarised in Table 2.

**Table 2.** Participant demographics

Participant^b^	Survey completed	Interview completed	Ethnicity	Age group	Sex
1 Alice	Yes	No	Black African	50–59	F
2 Jessica	Yes	Yes	Black African	60–69	F
3 Sophia	Yes	No	Black African	70–79	F
4 Mia	Yes	No	Black African	40–49	F
5 Faye	Yes	Yes	Black African	50–59	F
6 Karen	Yes	Yes	Black Caribbean	50–59	F
7 Laura	Yes	Yes	Black African	50–59	F
8 Hannah	Yes	No	Black Caribbean	60–69	F
9 Mandy	Yes	No	Black Caribbean	50–59	F
10 Emma	No	Yes	Black Caribbean	60–69	F
11 Ash	No	Yes	Black Caribbean	60–69	M

b Participant names are pseudonyms to retain participant anonymity.

### Key themes identified and their relevant domains

**Figure 1.**
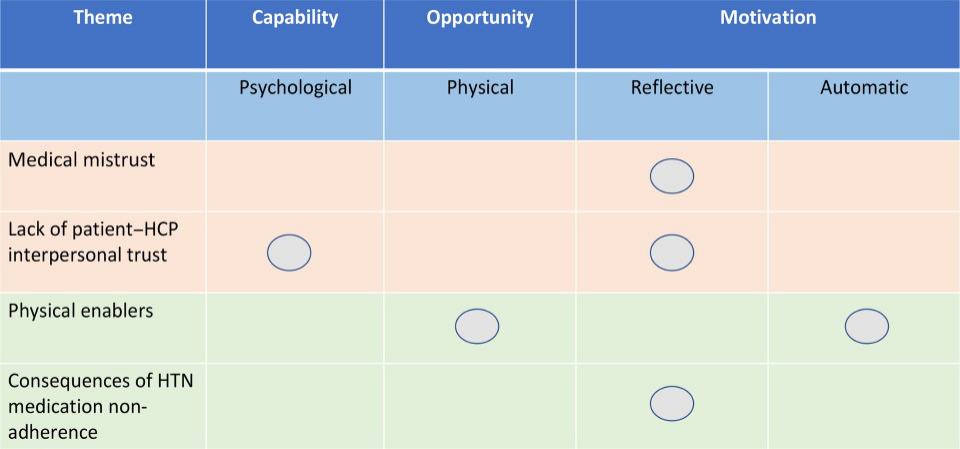
Themes reflecting barriers (in orange) and facilitators (in green) of prescribed medication for hypertension mapped onto the COM-B model constructs. COM-B = Capacity, Opportunity, and Motivation Behaviour model. HTN = hypertension. HCP = healthcare professional

Analysis drew four themes, two representing barriers and two representing facilitators to prescribed medication adherence for hypertension. Medical mistrust and lack of patient–HCP interpersonal trust emerged as barriers, while consequences of hypertension medication non-adherence and physical enablers were generated as facilitators. Participants provided more information for analysis contributing to barriers. All themes mapped to one or more of the following COM-B model constructs: psychological capability, physical opportunity, reflective motivation, and automatic motivation (see Table 3). Reflective motivation aligned to all themes except physical enablers and hence appeared the dominant construct in the analysis (see Figure 1).

**Table 3.** Qualitative themes mapped to COM-B model with a summary of the concepts contained within each element of the model

COM-B	Framework	Theme	Concepts	Barrier or facilitator
**Capability**	Psychological	Lack of patient–HCP interpersonal trust	Lack shared decision making, lack of patient-centred approach, time-poor consultations, not addressing medication concerns	Barrier
**Opportunity**	Physical	Physical enablers	Visual prompts, dosette box	Facilitator
**Motivation**	Reflective	Medical mistrust	Historical and widespread racial discrimination of the Black community, disproportionate morbidity and mortality rates, negative views of NHS and pharmaceutical companies	Barrier
		Lack of patient–HCP interpersonal trust	Limited discussion of lifestyle factors in addition to medication, lack of awareness of cultural approaches to medication diagnosis disbelief	Barrier
		Consequences of hypertension medication non-adherence	Fear of cardiovascular events, HCP role-modelling, failure of traditional (non-pharmacological) approaches	Facilitator
	Automatic	Physical enablers	Habit formation of taking medication regularly	Facilitator

COM-B = Capacity, Opportunity, and Motivation Behaviour model. HCP = Healthcare professional.

### Barriers

#### Medical mistrust

Participants described a sense of mistrust resulting from unequal treatment of the Black community that spanned beyond the relationship with any individual HCP and across the NHS. This context formed the basis of an active suspicion for medical intervention:

*'My feeling towards this is that we have an NHS who, for the main do a lot of good work, but I think as a community, I question whether the understanding of our needs are really taken into consideration … So I genuinely am distrustful of things that are supposedly looking to enhance things for us.'* (P11)

The interplay between socioeconomic position and mistrust of societal structures, including health care, gave further context to how some participants felt that poor provision of health care was in some way inevitable:

*'So we are very much a minority and I think as a general rule of thumb, those who are successful have to work doubly hard for it … So yeah, it catches up with you; long hours and the need to not fail … it’s the fact that … we're at the bottom of the heap in terms of housing, finance, jobs, etc.'* (P11)

Other participants indicated medical mistrust from the perspective of altered approaches taken towards them owing to being from the Black African or Black Caribbean ethnic group:

*'I think generally the way that Black people have been treated when it comes to health is mostly you're told, it’s not consultative.'* (P7)

Participants felt not only that they were spoken to differently but also health inequalities with respect to cardiovascular outcomes were described without explanation:

*'… I've been made to believe, you know, Black people suffer mostly with these thing; diabetes or blood pressure … Why is it that we are told that the number of Black people, they're more likely to be Black than White people are suffering with these things?'* (P10)

There was also a sense of frustration in the lack of understanding of hypertension as a diagnosis and its relation to vascular events:

*'Hypertension, I'm not even sure if stroke and hypertension are the same things.*' (P7)

There was scepticism of both the healthcare system and pharmaceutical companies and their perceived symbiotic relationship:

*'… in regards to pharmaceutical companies. I don't think with all due respect, that industry or the health system is here for getting people better … It’s far better to get you on a blood pressure pill followed by another pill and then there’s a side effect to that, which means you have to take something else, but big pharma is going, going and going …'* (P11)

#### Lack of patient–HCP interpersonal trust

Eight of eleven participants expressed sentiments related to interpersonal trust; this was particularly clear around the initiation of medication:

*'But yeah, there was no real discussion … It was just, you know, you take the pill and that’s it for ever more and a day.*' (P7)

There was a frustration among participants at the lack of consideration of more holistic management of hypertension by HCPs. Most participants mentioned benefits of lifestyle measures, such as diet and exercise, on managing hypertension:

*'I think you should be given the option to do the other things, but you know, it’s never been an option … before I started taking the medication I was already exercising … I would consider myself to be somewhat of a healthy person, I have a significantly healthy lifestyle.'* (P7)

Some participants displayed diagnosis disbelief reflecting a lack of trust in prescribed treatment:

*'I can do without it* [medication] *because actually there was a period when I completely forgot … By the time I reordered I had a gap you know and I didn't notice any difference. It didn't have any adverse effects.'* (P2)

The issue of concerns around medication side effects was prominent among five participants and descriptions of suboptimal communication and perceived impact regarding short consultations were described:

*'I was very unhappy to take any medication, as it’s foreign to our natural bodies, but I was not aware of alternative approaches at the time and there is lack of information regarding how safe it would be now to wean myself off the medication.'* (P8)

*'But in that 10 minutes, they're just going to give you the first thing that comes to mind … their first port of call is to give you some pills to get you to go away.'* (P7)

### Facilitators

#### Consequences of hypertension medication non-adherence

Concerns about not taking antihypertensive medication resulting in a stroke, heart attack, or acute hospital admission were cited by eight participants as motivating factors for taking their medication. Using fear-inducing tactics by HCP as a motivator was directly referred to:

*'She told me it’s bad, then I said, “I'm a nurse”, and the other people were probably thinking "wow, a nurse missing meds", I was embarrassed.'* (P5)

Being cognisant of the consequences of not taking hypertension medication was also shown as a driver to self-manage hypertension:

*'To be honest, I didn't take the tablets because how I grew up in the Caribbean, people don't take medication from doctors … they make up their own concoctions, get coconut water and squeeze a lemon in it … I was doing all those things that I heard people say but it got worse … It didn't work for me. I actually did need it.'* (P6)

### Physical enablers

All six interviewees mentioned using some form of physical enabler to assist them in taking their daily antihypertensive medication ranging from establishing routines, environmental triggers, or medicine compliance aids:

*'I'll leave it to where I can see it in the morning, because otherwise I can quite easily get up and start my day and I'll forget about taking it.*' (P10)

*'I've got a box in the kitchen which I keep in the cupboard, weekends I use that, then when I come to work, I use my dosette … I bought like a dosette box, a week one for seven days which I keep and I carry to work.'* (P5)

## Discussion

### Summary

This study has identified key themes that contribute to antihypertensive adherence in the Black community in South East London, which are related to trust: medical mistrust or lack of interpersonal trust with a HCP. It is pertinent to share these findings with HCPs locally and beyond to inform change within the systems that may reduce mistrust and reduce the inequality in cardiovascular outcomes.

Medical mistrust can be conceptualised as mistrust developed from past events and hierarchies that impact one’s relationships to organisations, communities, and other people, and acknowledges the impact of historical and current racial and cultural discrimination in the formation of medical mistrust in marginalised groups.^12^ Medical mistrust framed as group focused mistrust in those not belonging to the group, or of systems where they are ill-represented, usefully separates itself from interpersonal trust such as that between a patient and HCP.^13^ Medical mistrust emerged as participants expressed views on the challenges of system-wide discrimination across multiple sectors of housing, finance, and employment, and its potential impact on health and their perception of differential treatment from the NHS owing to their belonging to a Black ethnic group. These views are consistent with the *Birmingham and Lewisham African and Caribbean Health Inequalities Review (BLACHIR)* on inequalities in the borough of Lewisham, South East London,^14^ and other areas such as maternity services and COVID-19 vaccination.^15^

Interpersonal trust is an essential component of patient–HCP relationships and effective communication, confidentiality, and competence are key skills a physician requires to allow optimal interpersonal trust generation with a patient. Rolfe *at al* summarised patient trust as being earned through a belief that one’s best interests are being served by their doctor.^16^ The majority of participants expressed sentiments relating to patient–HCP interpersonal trust, including experiences of doctor-centric approaches, short consultations, inadequate addressing of medication concerns, and diagnosis disbelief, which were linked to distrust in HCPs.

It is logical that inequality as a driver of medical mistrust,^17^ owing to historical and ongoing racial or cultural discrimination, would lead to one assessing the motives of systems meant to improve their health negatively. Similarly, concerns over intentions of these systems could lead to decreased medication adherence, particularly if felt active harm could result. Medical mistrust aligns with the reflective motivation construct of COM-B, where plans and evaluations are made regarding whether to engage in a certain behaviour. Likewise, if experiences with HCPs have been suboptimal, interpersonal trust will be low leading to reflective motivation that discourages medication adherence.

HCP strategies perceived as inducing fear and HCP obligations to role model for patients were all elicited as aspects of motivation driving wants and needs, and subsequently evaluations that lead to plans to engage in the behaviour of taking antihypertensive medication. Consequences of not taking antihypertensive medication and their influencers align with the reflective motivation construct of COM-B as a facilitator of medication adherence with drivers of beliefs around benefit and values ascribed being a cognitive process of evaluation.

The participants using physical enablers all self-reported adherence, consistent with literature describing that intention and conscious decision making to adhere can be facilitated with practical physical solutions to support.^4^ Therefore, while physical enablers emerged as a facilitator to medication adherence among some participants, it must be recognised that this is dependent on their intention to adhere.

Participants using physical enablers, such as routines, environmental triggers, and medicine compliance aids, as facilitators to adherence corresponds to the physical opportunity construct of COM-B where cues and resources are utilised to assist in the behaviour of medication taking. Additionally, routines of medication taking that over time translate into habit also fit with automatic motivation as reflex responses to take tablets will occur when certain cues are triggered.

### Strengths and limitations

This study has generated insights that have been matched to a behavioural component within the narrative of medical management of hypertension; the reach of this study was increased through collaboration with community and public health organisations as well as interviews being performed by a local HCP of ethnic minority.

The biggest limitation to the study is the number of interviewees. There were initial challenges in reaching a reflective sample of people from the Black community in South East London, highlighting the challenges of recruitment in such qualitative studies and that those participating may not be truly representative of the population. For example, the majority of participants were female. Data saturation was not reached in this study, implying further interview and survey data collection could have enriched the analysis further. The material was primarily coded and analysed by first author NY who has a professional interest in quality improvement, is from a HCP background and has increased personal awareness of discrimination. In order to contrast the bias this may introduce to interpretation she was closely supervised by AB who is a non-clinical academic with a different ethnic background.

### Comparison with existing literature

The medical mistrust theme is aligned with recent literature expressing a pervasive sense of mistrust in organisations that shape determinants of health such as government,^17^ driven by inequalities faced by the Black community stemming from historical and current discrimination. Although trust barriers to antihypertension medication adherence have been identified in US studies, including African American participants,^18^ and medical mistrust has been described in the UK, in relation to reduced COVID-19 vaccine uptake,^19^ it has not, to the authors’ knowledge, been directly explored in people of Black Caribbean populations taking antihypertensive medication in London in the UK.

A qualitative study by Connell *et al*,^20^ consisting of 19 open-ended interviews with Black Caribbean patients with a diagnosis of hypertension, found beliefs that normal blood pressure readings did not require medication and practices of altering medication regimens based on symptoms believed to be associated with high blood pressure. This study also reported just under half of patients used herbal or bush remedies, with reluctance to discuss this with HCPs for fear of disapproval. It found the bulk of non-adherence was intentional and beliefs that hypertension was an acute problem with intermittent surges requiring treatment. This supports our findings that the binary division into adherent and non-adherent patients as unreflective of the complex and multifactorial issue of hypertension management for Black African and Black Caribbean individuals.

A recent study in Nepal using a similar methodology of inductive coding and deductive mapping to COM-B model illuminated culture-specific factors regarding hypertension as a condition that influenced adherence to high blood pressure medicines such as stigma associated with a diagnosis of hypertension, obesity reflecting positively on one’s wealth, and associations of low salt diets with bad luck,^21^ but has not been used to characterise behaviour associated with antihypertensive management in the UK.

### Implications for research and practice

The study outcomes have been disseminated to the Clinical Effectiveness South East London (CESEL) group, a primary care quality-improvement team working for the NHS South East London Integrated Care Board, who are responsible for tackling inequalities and improving outcomes for the local population. Intervention design focused on the barriers of trust and improving health literacy to fortify consequences of non-adherence are recommended to incorporate findings and cultural awareness training to minimise discrimination.

Since the completion of this research, findings have been added in combination with other insight work in South East London to local clinical guidelines on managing hypertension, produced predominantly for primary care to raise awareness of the effect of lack of interperonal trust and medical mistrust on hypertension care. Additionally, an 'anti-racism for health equity' community of practice has been set-up in Greenwich borough (South East London) led by NY, to improve cultural awareness and consider how to make anti-racist practices 'business as usual'.

Reflective motivation evaluations concluding to adhere to antihypertensive medication can be harnessed by improving patient–HCP interpersonal trust through interventions focused on encouraging HCPs to adopt patient-centred approaches incorporating shared decision making. Improving health literacy focused on: the asymptomatic nature of hypertension, potential of joint medication and lifestyle measures management strategies, addressing medication concerns, and clear explanation of hypertension risks including ethnicity-related risks. These types of interventions will also address the psychological capability construct of patient–HCP interpersonal trust as knowledge is required to support informed decision making regarding health choices.

## Data

The dataset relied on in this article is available from the corresponding author on reasonable request.
